# Supplementary material for: A fault-intrusion-tolerant system and deadline-aware algorithm for scheduling scientific workflow in the cloud
Source: PeerJ Comput Sci. 2021 Nov 2;7:e747. doi: 10.7717/peerj-cs.747 (PMC8576554; doi:10.7717/peerj-cs.747)
Supplement: Supplemental Information 6 [file peerj-cs-07-747-s006.zip › Myproject1.1/docs/org/cloudbus/cloudsim/examples/CloudSimExample8.html]

CloudSimExample8


JavaScript is disabled on your browser.


- Overview
- Package
- Class
- Use
- Tree
- Deprecated
- Index
- Help

- Prev Class
- Next Class

- Frames
- No Frames

- All Classes

- Summary:
- Nested |
- Field |
- Constr |
- Method

- Detail:
- Field |
- Constr |
- Method


org.cloudbus.cloudsim.examples

## Class CloudSimExample8

- java.lang.Object
- - org.cloudbus.cloudsim.examples.CloudSimExample8

- ---

    

  ```
  public class CloudSimExample8
  extends java.lang.Object
  ```

  An example showing how to create simulation entities
  (a DatacenterBroker in this example) in run-time using
  a globar manager entity (GlobalBroker).

- - ### Nested Class Summary

    Nested Classes

    | Modifier and Type | Class and Description |
    | --- | --- |
    | `static class` | `CloudSimExample8.GlobalBroker` |
  - ### Constructor Summary

    Constructors

    Constructor and Description

    `CloudSimExample8()`
  - ### Method Summary

    Methods

    | Modifier and Type | Method and Description |
    | --- | --- |
    | `static void` | `main(java.lang.String[] args)` Creates main() to run this example |

    - ### Methods inherited from class java.lang.Object

      `clone, equals, finalize, getClass, hashCode, notify, notifyAll, toString, wait, wait, wait`

- - ### Constructor Detail


    - #### CloudSimExample8

      ```
      public CloudSimExample8()
      ```
  - ### Method Detail


    - #### main

      ```
      public static void main(java.lang.String[] args)
      ```

      Creates main() to run this example


- Overview
- Package
- Class
- Use
- Tree
- Deprecated
- Index
- Help

- Prev Class
- Next Class

- Frames
- No Frames

- All Classes

- Summary:
- Nested |
- Field |
- Constr |
- Method

- Detail:
- Field |
- Constr |
- Method
